# Efficient Blue-emitting Phosphor SrLu_2_O_4_:Ce^3+^ with High Thermal Stability for Near Ultraviolet (~400 nm) LED-Chip based White LEDs

**DOI:** 10.1038/s41598-018-28834-8

**Published:** 2018-07-11

**Authors:** Sheng Zhang, Zhendong Hao, Liangliang Zhang, Guo-Hui Pan, Huajun Wu, Xia Zhang, Yongshi Luo, Ligong Zhang, Haifeng Zhao, Jiahua Zhang

**Affiliations:** 1 State Key Laboratory of Luminescence and Applications, Changchun Institute of Optics, Fine Mechanics and Physics, Chinese Academy of Sciences, 3888 Eastern South Lake Road, Changchun, 130033 China; 2 University of Chinese Academy of Sciences, Beijing, 100049 China

## Abstract

Blue-emitting phosphors for near ultraviolet (NUV) based tri-color RGB phosphor blend converted white light emitting diodes (LEDs) have been extensively investigated in the past few years. LED chip peaked near 400 nm is the most efficient among the NUV chips currently. However, most of blue phosphors show inefficient excitation around 400 nm. Herein, a novel blue phosphor SrLu_2_O_4_:Ce^3+^ matching well with near 400 nm chip and showing high thermal stability has been developed. The photoluminescence spectrum presents a broad emission band peaking at 460 nm with a bandwidth of nearly 90 nm. By optimizing the Ce^3+^ concentration, an internal quantum efficiency (IQE) as high as 76% was achieved. Furthermore, 86% of the room-temperature emission intensity is still maintained at 150 °C, indicating a good thermal stability and practicality. A series of white LEDs were fabricated based on 405 nm chips coated with a blend of the new blue phosphor with the commercial yellow and red phosphors. High color rendering indexes (≥90) were achieved while the correlated color temperature was tuneable in the range of 3094 to 8990 K. These results suggest that SrLu_2_O_4_:Ce^3+^ can be utilized as a blue-emitting phosphor in NUV based white LEDs.

## Introduction

White LEDs have been widely deployed commercially on solid-state lighting, because of their superior efficiency and long life^1^. Up to now, the most commonly used method to get white light based on monochrome LED is to combine a yellow-emitting phosphor such as cerium substituted yttrium aluminum garnet (Y_3_Al_5_O_12_: Ce^3+^) with a blue (InGa)N LED chip^2^. However, the inconsistency of the aging characteristics of the blue chip and the phosphor can lead to the instability of the white light which is generated by the combination of the blue light from the chip and the yellow light from the phosphor. Furthermore, this type of white LED emits little red light and therefore has a low color rendering index^3–8^. To resolve this problem, one can use an alternative method to obtain stable white light by a combination of a red-green-blue (RGB) phosphor blend with a near ultraviolet (NUV) LED chip^9,10^. In this method, the visible components of the white light are generated only by phosphors, exhibiting low color point variation against the forward-bias currents. In the NUV chips, near 400 nm chip is more attractive because of the highest energy conversion efficiency. As a result, this approach requires all three RGB phosphors to have efficient excitation around 400 nm to maximize the device’s efficiency.

Some blue-emitting phosphors as candidates for NUV (~ 400 nm) chip excitation have been reported, such as LiCaPO_4_: Eu^2+^ ^11^, Ba_3_LaNa(PO_4_)_3_F: Eu^2+^ ^12^, BaHfSi_3_O_9_: Eu^2+^ ^13^, Ca_2_LuScZrAl_2_GeO_12_: Ce^3+^ ^14^, Ca_3_Hf_2_SiAl_2_O_12_: Ce^3+^ ^15^, Ca_3_Zr_2_SiGa_2_O_12_: Ce^3+^ ^16^, CaLaGa_3_S_6_O: Ce^3+^ ^17^, Ca_2_B_5_O_9_Br: Eu^2+^ ^18^, and Na_3–2x_Sc_2_(PO_4_)_3_: xEu^2+^ ^19^. But few of them could match well with near 400 nm chip and have high thermal stability at the same time. Therefore, it is essential to develop novel blue-emitting phosphors showing both excellent thermal stability and high quantum efficiency.

The rare-earth strontium oxides, SrLn_2_O_4_ (where Ln = Gd, Ho, Er, Tm, Yb), with the space group *Pnam* have been intensively studied for their magnetic properties^20–22^. Selected SrLn_2_O_4_ as hosts, Eu^3+^-doped SrLu_2_O_4_ and SrGd_2_O_4_ luminescent materials have been studied and considered as promising red phosphors in solid state lighting devices^23,24^. Eu^2+^ doped SrLu_2_O_4_ exhibited a broad red emission centered at 610 nm, but it could be applied in optical temperature sensor instead of white LED because of its strong thermal quenching^25^.

In this paper, we report to our knowledge for the first time, a novel blue phosphor of SrLu_2_O_4_: Ce^3+^ prepared by solid state reaction. Photoluminescence (PL) properties and temperature dependence of the new phosphor are studied. The new phosphor shows efficient blue emission with the luminescence excitation band matching well with near 400 nm NUV chip. Besides, the phosphor also has high thermal stability with 86% of its room temperature emission intensity remained at 150 °C. White LEDs with high color rendering index (CRI) at different correlated color temperature (CCT) were fabricated based on NUV chips coated with blends of the new blue phosphor with the commercial yellow and red phosphors.

## Results and Discussion

**Figure 1:**
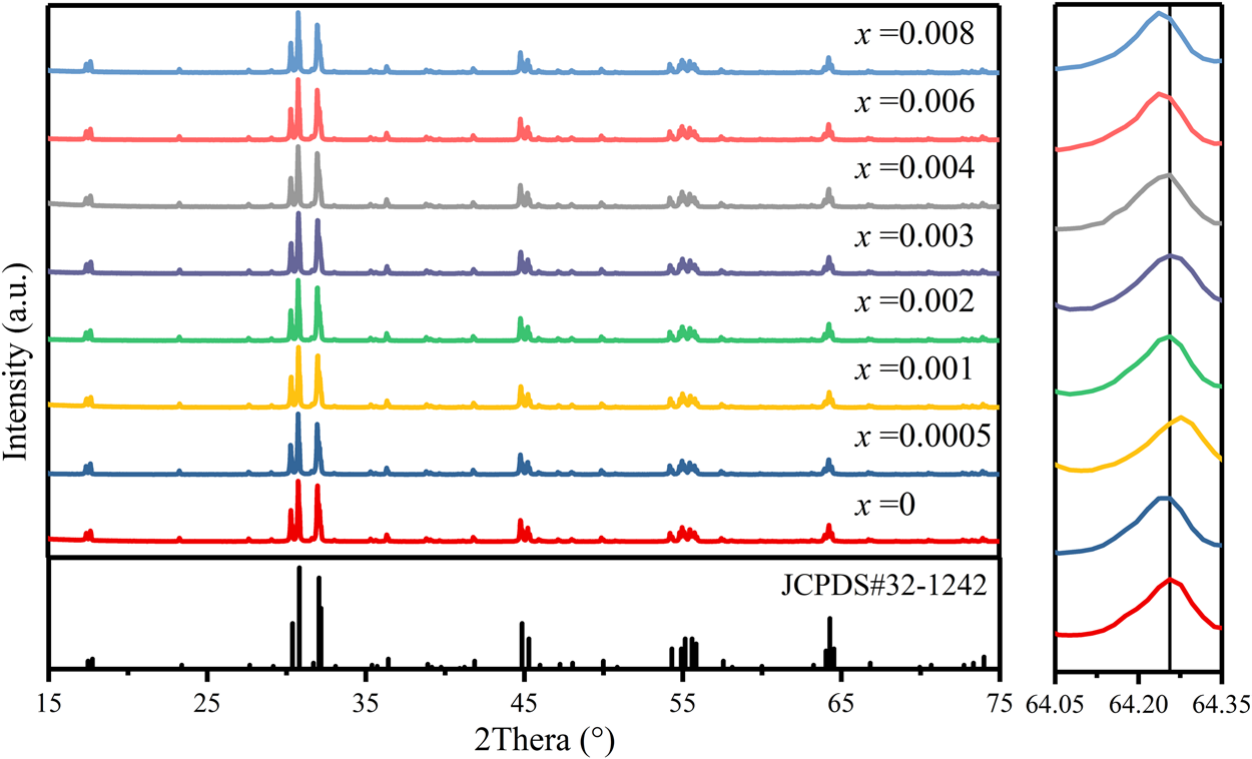
XRD patterns of prepared SrLu_2−*x*_O_4_: *x*Ce^3+^(*x* = 0.0005-0.008) phosphors. As a reference, the standard XRD pattern of SrLu_2_O_4_ (JCPDS No. 32-1242) is included. Right inset shows the peak shift of series XRD patterns with reference line locating at 64.2565°.

The phase purities and the crystal structures of the as-prepared powder samples SrLu_2-*x*_O_4_: *x*Ce^3+^(*x* = 0.0005-0.008) were characterized by XRD at room temperature. These samples all exhibit a single phase similar with the SrLu_2_O_4_ (JCPDS# 32-1242) crystal structure in Fig. 1. It is clearly that no new impurity appeared with the increasing concentration of Ce^3+^. A slight shift of peak at 64.3° can be noticed. When *x* equals 0.01, peak shifts to bigger angle and it shifts to minor angle while *x* is more than 0.004. It can also be explained by Bragg equation:

1 $$2\mathrm{dsin}{\rm{\theta }}=n{\rm{\lambda }}$$

Peak shifting to minor angle means sites are occupied by bigger ions. In SrLu_2_O_4_ structure, the ion radius of Ce^3+^(CN = 6, r = 1.01 Å; CN = 8, r = 1.143 Å) is between Sr^2+^(CN = 8, r = 1.26 Å) and Lu^3+^(CN = 6, r = 0.861 Å) which means it might take both sites. At low concentrations, Ce^3+^ tend to occupy Sr^2+^ sites. When *x* equals 0.01, Ce^3+^ occupied more Sr^2+^ than Lu^3+^ sites and represented shifting to bigger angle. While *x* is more than 0.004, Ce^3+^ occupied more Lu^3+^ than Sr^2+^ sites and represented shifting to minor angle. In this work, all the samples were synthesized based on the substitution of Ce^3+^ for Lu^3+^ by charge balance. However, it does not follow that the Ce^3+^ only occupies the Lu^3+^ site as mentioned above.

**Figure 2:**
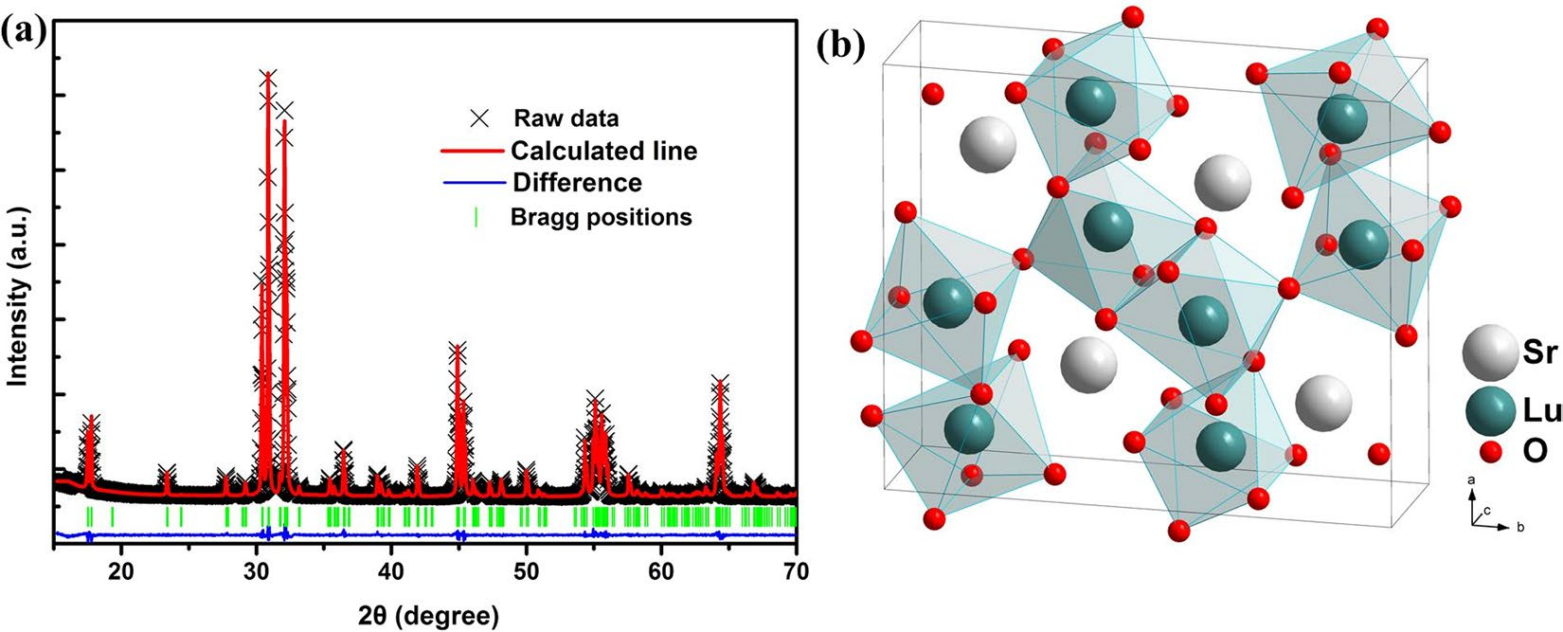
(**a**) Rietveld refinements for SrLu_2_O_4_. (**b**) Crystal structure schematic diagram of SrLu_2_O_4_.

In order to further understand the microstructure of the as-prepared samples, detailed Rietveld refinements and lattice parameters are performed in Fig. 2(a) and Table 1. The crystal structure schematic diagram of SrLu_2_O_4_ is obtained according to the JCPDS cord using Diamond software, shown in Fig. 2(b). The Sr site is 8-coordinated with average Sr-O bond length of 2.6145 Å, while Lu site is 6-coordinated with average Lu-O bond length of 2.2917 Å.

**Table 1:** Rietveld refinement data of SrLu_2_O_4_.

Formula	SrLu_2_O_4_
Temperature/K	300 K
Space group	*Pnam*(64)
Z	12
**Lattice parameters**	
a/Å	9.9747
b/Å	11.7482
c/Å	3.3395
V/Å^3^	391.34
R_p_/%	5.76
R_wp_/%	6.35

**Figure 3:**
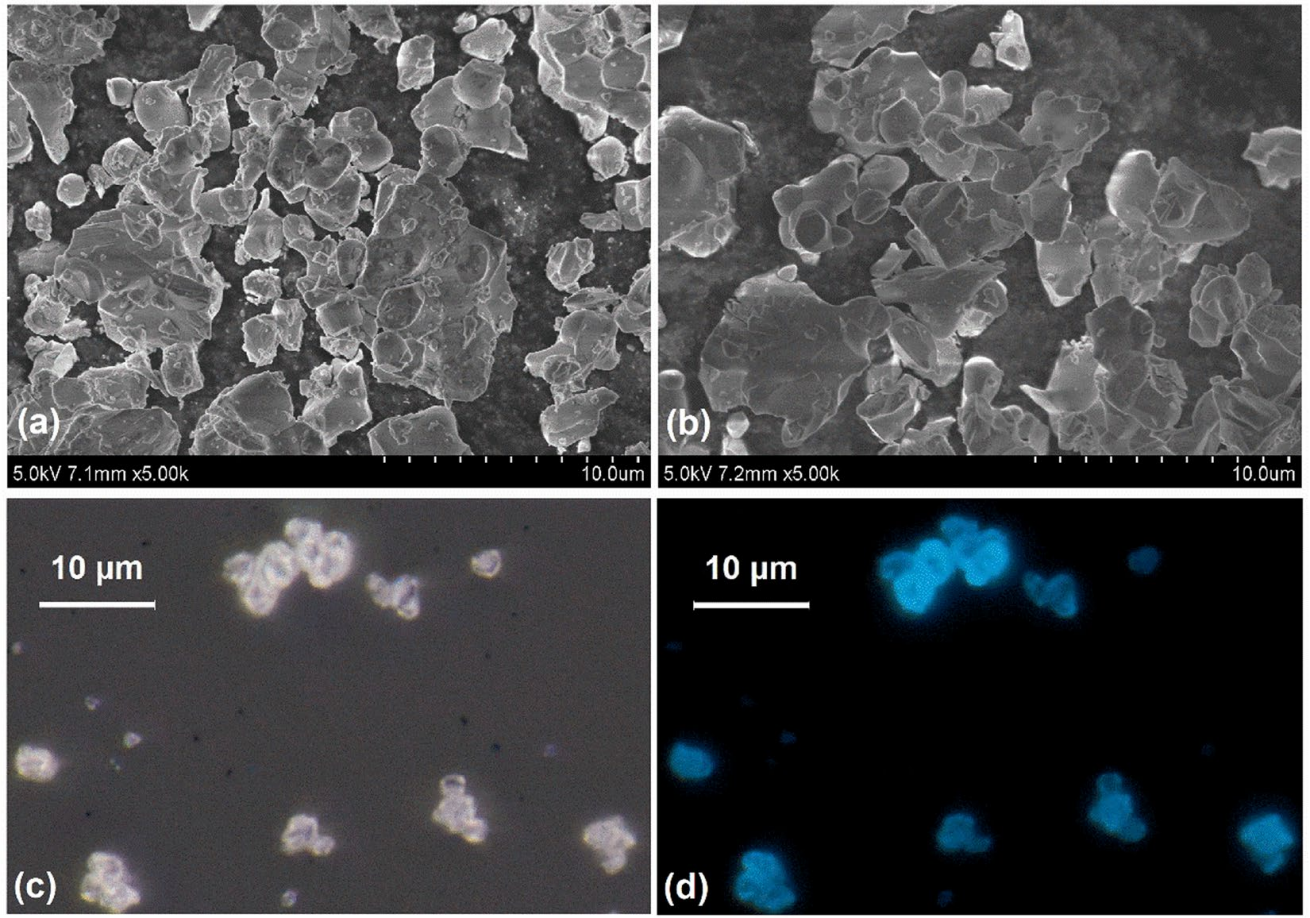
SEM images of SrLu_2_O_4_: Ce^3+^ phosphors unwashed (**a**) and washed (**b**). The photos of SrLu_2_O_4_: Ce^3+^ under fluorescence microscopy with bright field light (**c**) and 405 nm light (**d**) excitation.

Figure 3(a) and (b) show the SEM images of SrLu_2_O_4_: 0.2% Ce^3+^ unwashed and washed, respectively. It can be seen that small particles were washed away by alcohol and the particle was about 3 µm. Figures 3(c,d) show the micrographs of SrLu_2_O_4_: 0.2% Ce^3+^ phosphors under bright field light and 405-nm excitation, respectively. The size of particles in micrographs agrees with those in SEM images.

**Figure 4:**
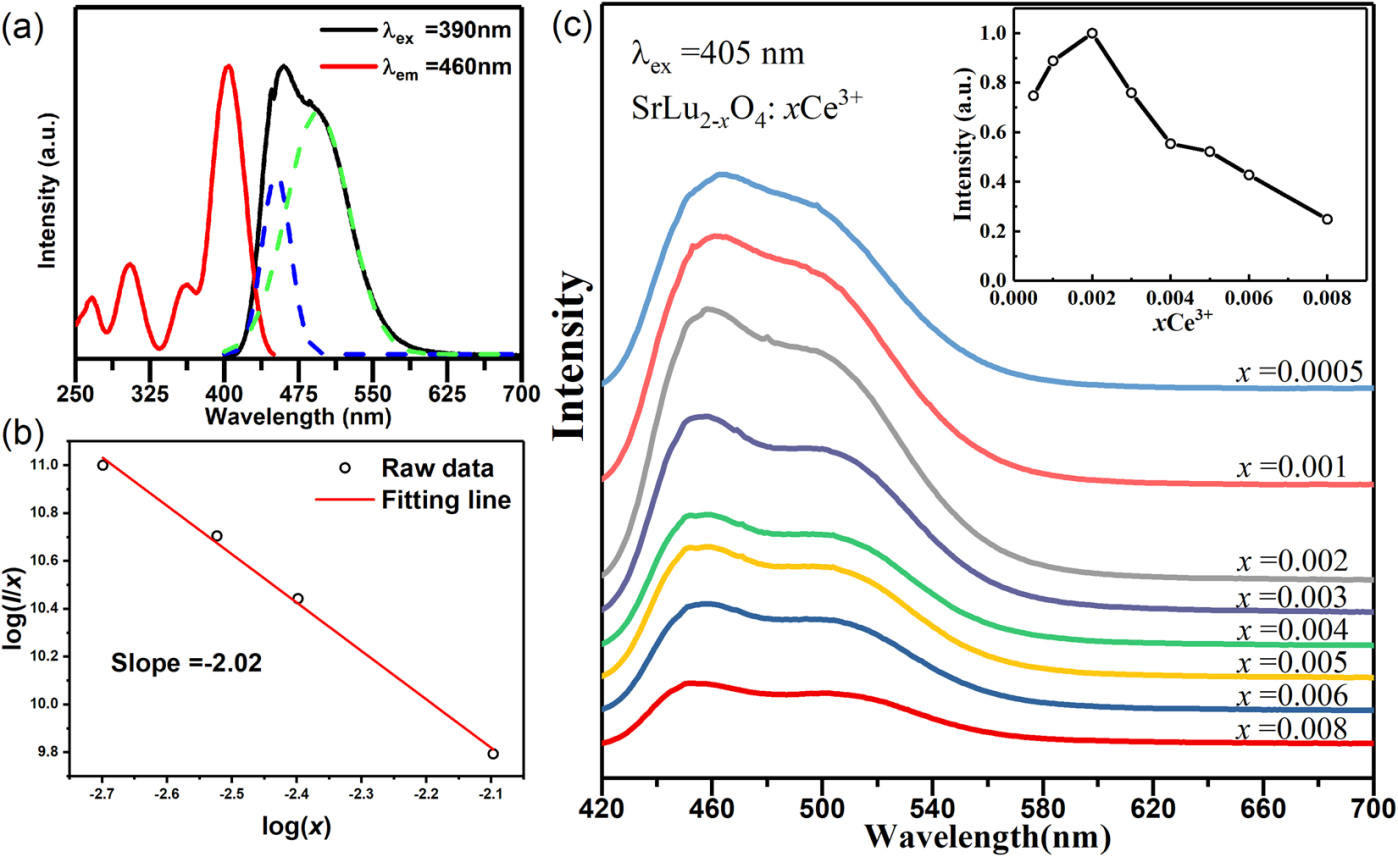
(**a**) PLE and PL spectra of SrLu_1_._998_O_4_: 0.002Ce^3+^ with the emission band fitted with two Gaussian curves (dashed). (**b**) Linear fitting of log(*x*) versus log(*I/x*) in the SrLu_2-x_O_4_: *x*Ce^3+^ samples. (**c**) PL spectra of SrLu_2-x_O_4_: *x*Ce^3+^(*x* = 0.0005-0.008) under 405 nm excitation, the inset shows the dependence of integrated emission intensities on Ce^3+^ concentration.

Figure 4(a) shows the normalized PLE (λ_em_ = 460 nm) and PL (λ_ex_ = 390 nm) spectra of SrLu_2_O_4_: 0.2% Ce^3+^ phosphors. The PLE spectrum of SrLu_2_O_4_: Ce^3+^ in the UV region from 250 to 450 nm contains four distinctive bands peaked at 265 nm, 306 nm, 359 nm and 405 nm mainly ascribed to the allowed 4*f*-5*d* transitions of Ce^3+^ ^26^. To obtain entire PL spectrum, we chose 390 nm as the excitation wavelength to avoid the effect of excitation light. The PL spectrum exhibits a strong blue emission peak at 460 nm with a full width at half maximum (FWHM) of approximately 90 nm. The asymmetric emission band can be well fitted with two Gaussian bands peaking at 20253 cm^−1^ and 22165 cm^−1^. Their energy difference is about 1912 cm^−1^, being consistent with the energy separation between the ^2^F_7/2_ and the ^2^F_5/2_ sub-states of the ground state of Ce^3+^ (~2000 cm^−1^)^27^.IQEs were measured using a spectral on-coated integrating sphere and the measured results show that the IQE is 76.1%. The optimization of Ce^3+^ concentration in SrLu_2_O_4_ was performed by studying the PL spectra of SrLu_2−*x*_O_4_: *x*Ce^3+^ for different Ce^3+^ concentrations (*x* = 0.0005, 0.001, 0.002, 0.003, 0.004, 0.006 and 0.008), as shown in Fig. 4(c). The concentration dependence of the PL intensities is depicted in the inset. It is clear that the maximal PL intensity occurs around 0.2% Ce^3+^. Further increasing Ce^3+^ concentration leads to PL decreasing due to the well-known concentration quenching effect. The concentration quenching originates from the larger probability of energy loss at a killer centre due to excitation energy migration among Ce^3+^ ions.

Here, the critical quenching distance (R_c_), defined as an average distance between Ce^3+^ ions shorter than which luminescence quenching occurs, can be estimated from geometrical consideration by following formula^5,28^:

2 $${{\rm{R}}}_{c}\approx 2{[\frac{3V}{4\pi {X}_{c}N}]}^{1/3}$$

where V represents the volume of the unit cell, X_c_ represents the critical concentration of Ce^3+^ ions, and N represents the number of sites that can be replaced by Ce^3+^ in the unit cell. In this study, V = 391.339 Å^3^, N = 12, and X_c_ = 0.002 for SrLu_2_O_4_: Ce^3+^. Thus, the R_c_ of Ce^3+^ ions were calculated to be 31.46 Å. The value is much larger than 5 Å, indicating that exchange interaction is not responsible and the electric multipolar−multipolar interaction should be suitable for the ET of this luminescence center^29^.

The energy transfer mechanism among Ce^3+^ ions in this system is governed by electric multipolar interactions based on the Dexter theory. Furthermore, according to the report of Van Uitert the PL intensity and activator concentration satisfy the following equation^30^.

3 $$\frac{I}{x}=\frac{k}{1+\beta {(x)}^{1/\theta }}$$

where *I* is the PL intensity, *x* is the activator ion concentration, which is not less than the critical concentration, *k* and *β* are constants for a given host crystal under the same excitation conditions, and *θ* is an indication of the type of electric multipolar interactions. The value of *θ* is 6, 8, and 10, standing for the energy transfer mechanism of electric dipole-dipole, dipole-quadrupole, and quadrupole-quadrupole interactions, respectively. As shown in Fig. 4(b), the relationship of log(*I/x*) versus log(*x*) can be fitted linearly with a slope −(*θ/3*) equal to −2.02. The value of *θ* is determined to be 6.06, which approximates to 6, implying that the concentration quenching of Ce^3+^ ions in the SrLu_2_O_4_: Ce^3+^ mainly results from the electric dipole-dipole interactions between Ce^3+^ ions. The large critical quenching distance might be explained as follows. Ce^3+^ ions may occupy Sr^2+^ and Lu^3+^ sites to form two luminescence centers (Ce1 and Ce2). We found that there was another emission band centered at 600 nm upon 480-nm excitation, as shown in Figures S1,S2. Here, we name the new band Ce2. One can observe that the PLE band centered at 485 nm of Ce2 entirely overlaps with the PL band of Ce1, resulting in effective energy transfer from Ce1 to Ce2. The appearance of the strong Ce1 PLE band at 390 nm in the PLE spectrum of Ce2 is strong evidence for effective energy transfer. However, the Ce2 emission is too weak to be discernible compared to Ce1 center under 405-nm excitation at room temperature, as shown in Figure S3. This is because Ce2 is thermally quenched at room temperature. The detail study of luminescent properties of Ce2 is in progress. In this work, we lay emphasis on the optical properties of Ce1 center. The large critical distance for luminescence quenching of Ce1 is, therefore, attributed to effective energy transfer from Ce1 to Ce2.

**Figure 5:**
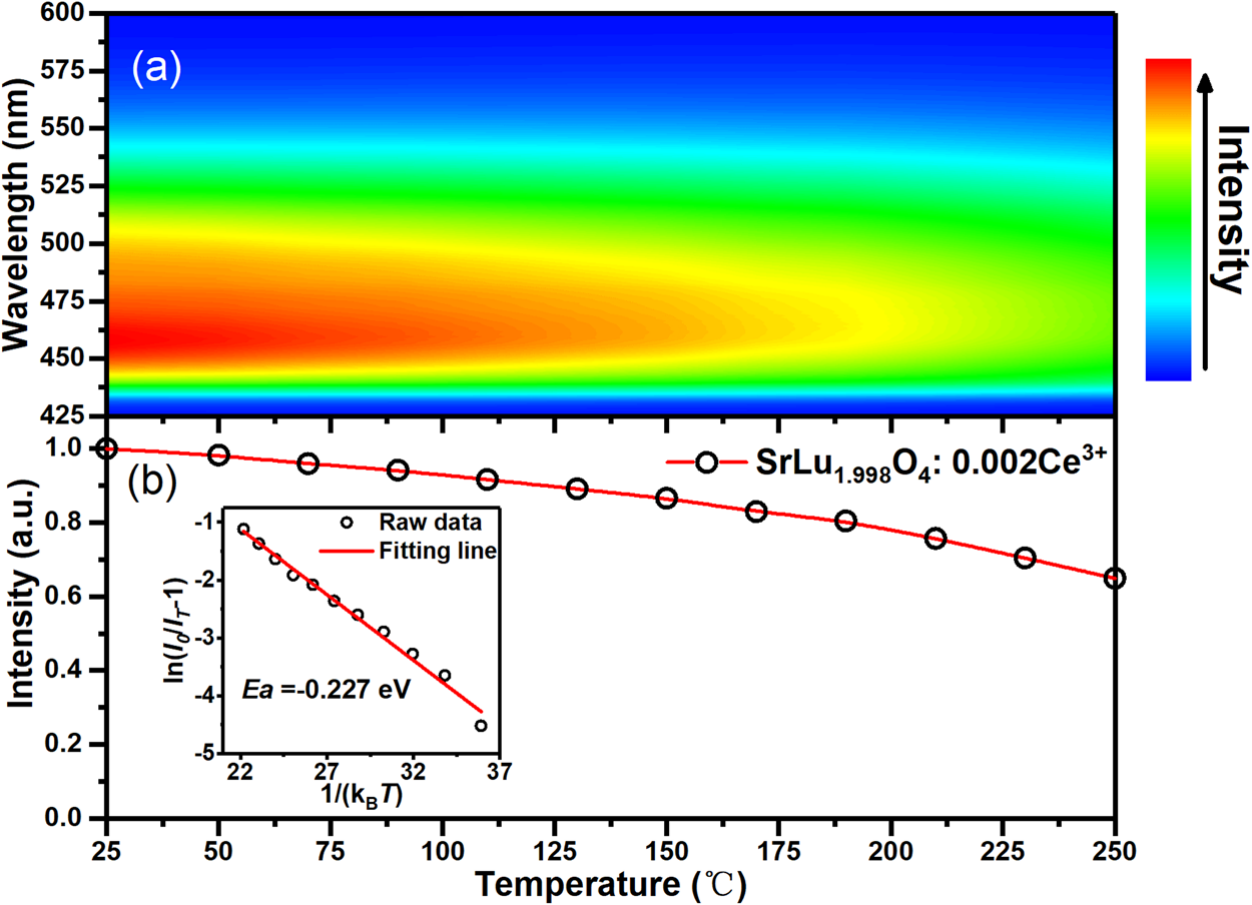
(**a**) Temperature-dependent measurement of the emission spectrum of SrLu_1_._998_O_4_: 0.002Ce^3+^. (**b**) Temperature dependence of the integrated emission intensities in SrLu_1_._998_O_4_: 0.002Ce^3+^ excited at 405 nm. The inset shows the Arrhenius fitting of the emission intensities.

The temperature-quenching property of SrLu_1_._998_O_4_: 0.002Ce^3+^ was also studied. This new blue phosphor, SrLu_1_._998_O_4_: 0.002Ce^3+^, shows a stability in the luminescence color. As shown in Fig. 5(a), a slight red shift and decrease in emission in intensity can be observed with increasing temperature. In Fig. 5(b), the emitting intensity was up to 90% below 120 °C and still above 60% at 250 °C of that at room temperature (25 °C). It shows respectable thermostability and the luminescence properties of some recent blue phosphors are listed on Table 2. To better understand the temperature dependence of photoluminescence, the activation energy was calculated using the Arrhenius equation given as^31,32^

4 $$I(T)=\frac{{I}_{0}}{1+A{e}^{-{E}_{a}/{k}_{b}T}}$$

where *I*_*0*_ is the PL intensity at 0 K, here it is treated as the one at room temperature if the PL intensity is stable below room temperature, *I(T)* is the PL intensity at a given temperature *T*, *A* is a constant, *E*_*a*_ is the activation energy for thermal quenching, and *k*_*B*_ is the Boltzmann constant. The experimental data are well fitted using Eq. (4), as shown in inset in Fig. 5(b). The value of *E*_*a*_ was obtained to be 0.227 eV for SrLu_1_._998_O_4_: 0.002Ce^3+^. As the heat-treatment temperature increases, the thermal quenching of luminescence is usually attributed to a thermal activation process in which the excited electron energy is released preferentially through heat dissipation by phonons rather than radiation by photons.

**Table 2:** Comparation of luminescence properties of SrLu_2_O_4_: Ce^3+^ and some existing blue phosphors. *I*_150 °C_ denotes the luminescence intensity or QE at 150 °C relative to the value at RT.

Phosphor formula	λ_ex_/nm	λ_em_/nm	*I*_150 °C_ (%)	ΔE/eV	Ref.
SrLu_2_O_4_:Ce^3+^	385-425	460	86	0.227	this work
Ca_5_._45_Li_3_._55_(SiO_4_)_3_O_0_._45_F_1_._55_:Ce^3+^	330-420	470	71	0.27	^4^
Ca_1_._65_Sr_0_._35_SiO_4_:Ce^3+^	270-380	450	73	0.179	^33^
K_2_ZrSi_3_O_9_:Eu^2+^	380-420	465	35	0.304	^34^
YScSi_4_N_6_C:Ce^3+^	280-425	469	48	0.334	^35^
Ba_9_Lu_2_Si_6_O_24_:Eu^2+^	250-420	460	39.6	0.341	^36^

**Figure 6:**
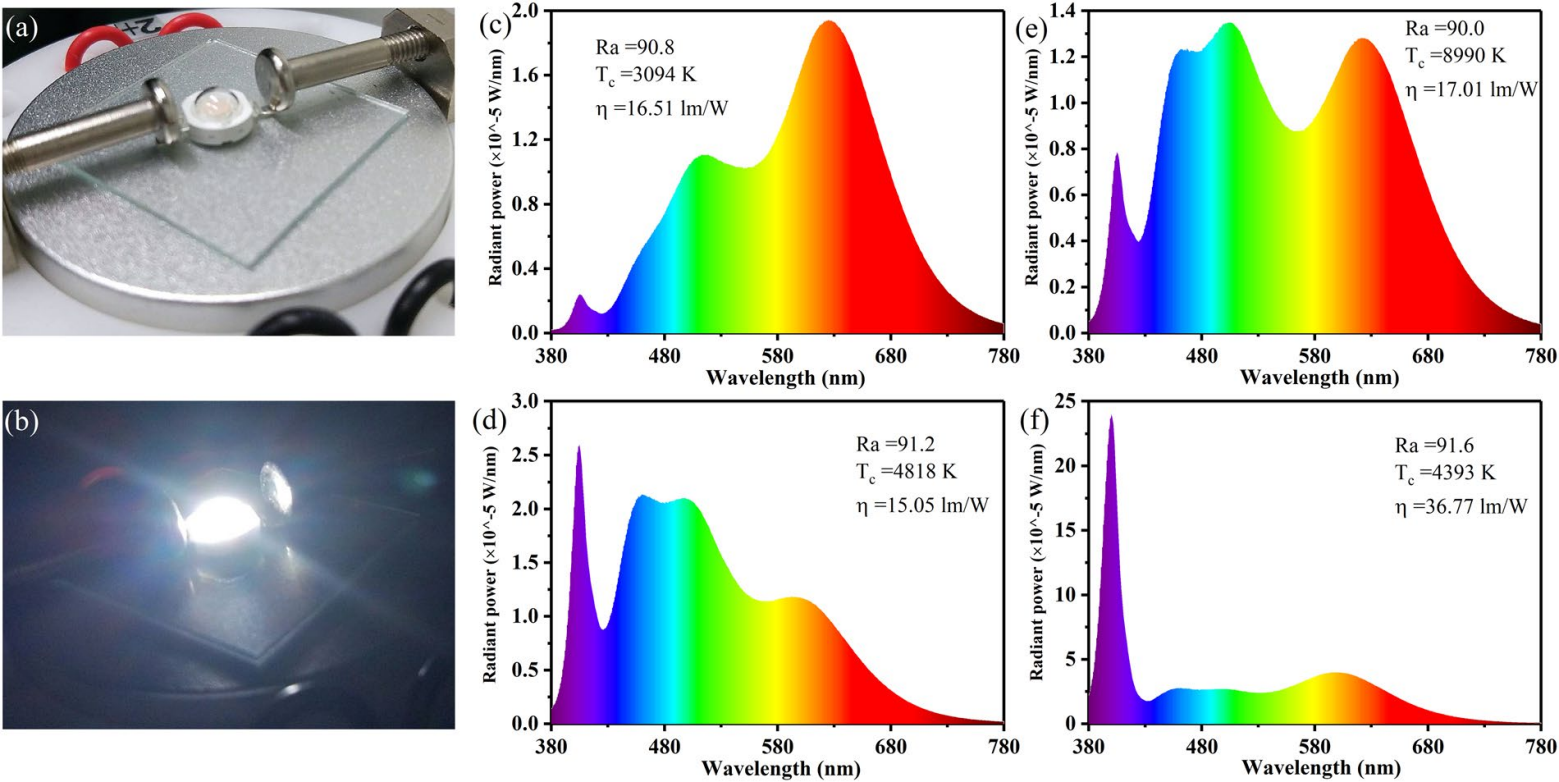
Digital images of the white LED unworked (**a**) and working under 20 mA and 3 V (**b**). (**c**–**f**) Emission spectra of white LEDs fabricated using NUV 405-nm chips combined with different ratios of SrLu_2_O_4_: Ce^3+^ (blue), (Sr, Ba)_2_SiO_4_: Eu^2+^ (yellow) and Sr_2_Si_5_N_8_: Eu^2+^ (red) phosphors under a forward bias of 20 mA and 3 V.

Combined with SrLu_2_O_4_: Ce^3+^ (blue), (Sr, Ba)_2_SiO_4_: Eu^2+^ (yellow) and Sr_2_Si_5_N_8_:Eu^2+^ (red) phosphors, the white LEDs based on NUV 405-nm chips have been fabricated and the digital images are shown in Fig. 6(a) and (b). Figure 6(c–f) show the electroluminescent spectra of fabricated white LED lamps with high color rendering index (Ra ≥ 90). By changing different ratio of constituents, different correlated color temperature (CCT) can be achieved maintaining high Ra and the detailed parameters were listed in Table 3. White LEDs (c-e) were fabricated preliminarily to get high Ra and the luminous efficiency was overlooked. After fabrication optimization, considerably enhanced luminous efficiency of 36.77 lm/W with 91.6 Ra was achieved in white LED (f). The white LED luminous efficiency will be greatly improved in the future after fabricating conditions optimization in all aspects.

**Table 3:** Performance parameters of four white LEDs under the current of 20 mA.

White LED	Weight ratio of constituents (%)			Ra	CCT (K)	η (lm/W)	CIE coordinates	
	blue	yellow	red				x	y
(c)	98.36	0.66	0.98	90.8	3094	16.51	0.4354	0.4125
(d)	98.52	0.49	0.99	91.2	4818	15.05	0.353	0.3774
(e)	98.93	0.25	0.82	90.0	8990	17.01	0.2783	0.3163
(f)	98.46	0.56	0.98	91.6	4393	36.77	0.3582	0.3343

**Figure 7:**
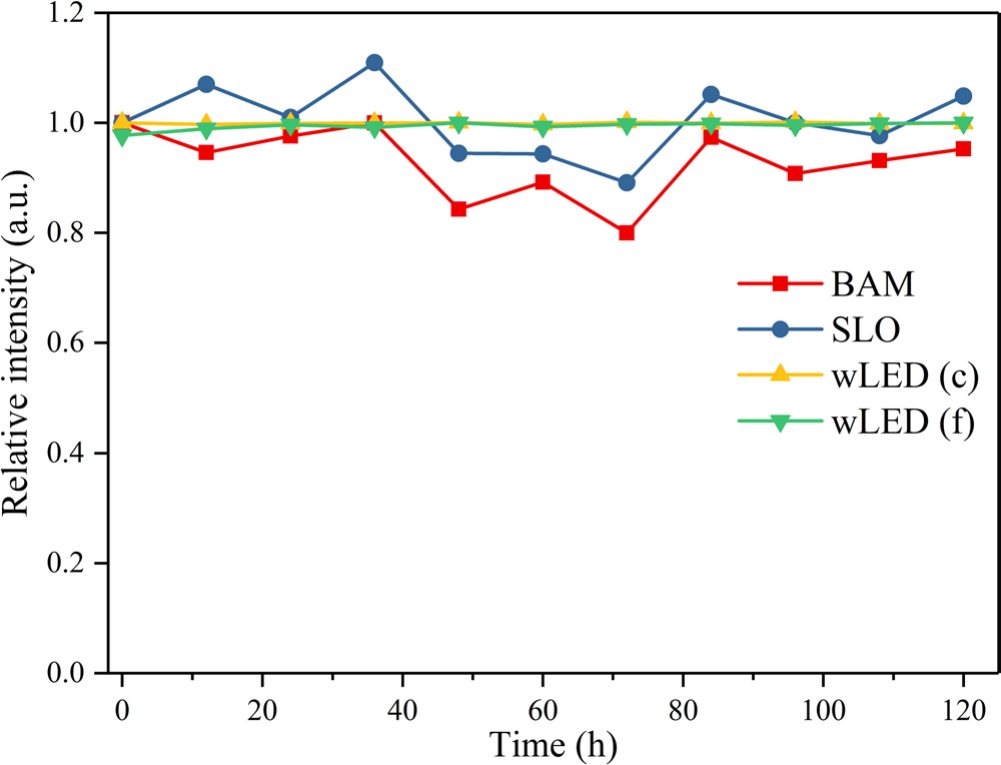
Relative integrated intensity of powder phosphors and fabricated white LEDs as a function of time: at temperature of 85 °C, in humidity at 85% RH.

We chose SrLu_1_._998_O_4_:0.002Ce^3+^, commercial blue phosphor BAM and fabricated white LEDs (c, f) to carry on the conventional high temperature/high humidity (85 C/85RH) tests to prove its reliability. As shown in Fig. 7, white LEDs showed a great stable locating at 1.0 while both SLO and BAM phosphors fluctuated around 1.0 and SLO was better. Another group of fabricated white LEDs with near 40 lm/W were acquired and carried on the reliability test in Figures S4,S5, which also show great reliability.

All these results indicate that SrLu_2_O_4_: Ce^3+^ phosphors have promising applications for white-light NUV LEDs.

## Conclusions

The Ce^3+^ doped SrLu_2_O_4_ phosphor was synthesized via high-temperature solid-state reaction method. The XRD patterns indicated the purity of the crystal phase for the as-prepared samples. Under UV excitation at 405 nm, the PL spectrum of SrLu_1_._998_O_4_: 0.002Ce^3+^ sample exhibits a broad blue emission band peaked at 460 nm with a high IQE of 76.1%. By changing the ratios of SrLu_2_O_4_: Ce^3+^, yellow-emitting (Sr, Ba)_2_SiO_4_: Eu^2+^, and red-emitting Sr_2_Si_5_N_8_: Eu^2+^ phosphors, a series of white NUV LEDs with excellent color rendering index (Ra ≥ 90) could be fabricated, and the correlated color temperatures were 3094 K, 4393 K, 4818 K and 8990 K, respectively. All results indicate that SrLu_2_O_4_: Ce^3+^ can be promisingly used as an ultraviolet-convertible blue-emitting phosphor.

## Methods

### Materials Synthesis

SrLu_2_O_4_: Ce^3+^ samples were synthesized by traditional high-temperature solid-state reaction. The constituent oxides and carbonates, Sr_2_CO_3_ (A.R.), Lu_2_O_3_ (99.99%), CeO_2_ (99.99%) were employed as the raw materials. The stoichiometric weighed powder according to the formula of SrLu_2-*x*_O_4_: *x*Ce^3+^ was mixed in an agate mortar and placed in an alumina crucible. This crucible was heated at 1600 °C for 6 hours in a reducing atmosphere (95%N_2_/5%H_2_), to reduce Ce^4+^ to Ce^3+^. After sintering, the powders were furnace-cooled naturally down to room temperature (RT). Finally, the as-prepared powders were washed with alcohol three times and dried at 60 °C for 6 hours in a drying oven to obtain final phosphors. The NUV 405-nm chips we used were produced by Guangsheng Semiconductor Technology Co., Ltd. (chip size is 1.143 mm × 1.143 mm and efficiency is 40%)

### Characterization

X-ray diffraction (XRD) patterns were performed by a powder diffractometer (Bruker, D8 Focus, Cu Kα, 40 kV, 40 mA). The XRD data were collected in range of 15 to 75 degree (2θ) with count time of 2 s/step. The PL and photoluminescence excitation (PLE) spectra of Ce^3+^ were measured by FL900 fluorometer with a Xenon lamp (Edinburg Instruments, UK). The fluorescence micrographs and temperature-dependent PL spectra were carried out on a fluorescence microscopy (Olympus, BX53M). Internal quantum efficiency (IQE), i.e. the number ratio of the photon emitted to the photon absorbed, were measured directly by the absolute PL quantum yield measurement system (C9920-02, Hamamatsu Photonics K.K., Japan). The high temperature/high humidity (85 C/85RH) condition was provided by Programmable Temperature & Humidity Chamber (BPHJS-060A, China). The photoelectric properties of the fabricated white LEDs were measured by HAAS 2000 photoelectric measuring system (380 nm–780 nm, EVERFINE, China). The forward bias current was 20 mA. All the measurements were conducted at room temperature unless mentioned specially.

## Electronic supplementary material

Supplementary Information
