# Supplementary material for: Efficient Blue-emitting Phosphor SrLu2O4:Ce3+ with High Thermal Stability for Near Ultraviolet (~400 nm) LED-Chip based White LEDs
Source: Sci Rep. 2018 Jul 11;8:10463. doi: 10.1038/s41598-018-28834-8 (PMC6041296; doi:10.1038/s41598-018-28834-8)
Supplement: Supplementary file 1 — Supplementary Information [file 41598_2018_28834_MOESM1_ESM.docx]

Supplementary Information for

“Efficient Blue-emitting Phosphor SrLu_2_O_4_:Ce^3+^ with High Thermal Stability for Near Ultraviolet (~ 400 nm) LED-Chip based White LEDs”

Sheng Zhang,^a,b^ Zhendong Hao,^a,*^ Liangliang Zhang,^a^ Guo-Hui Pan,^a^ Huajun Wu,^a^ Xia Zhang,^a^ Yongshi Luo,^a^ Ligong Zhang,^a^ Haifeng Zhao,^a^ Jiahua Zhang.^a,*^

^a^State Key Laboratory of Luminescence and Applications, Changchun Institute of Optics, Fine Mechanics and Physics, Chinese Academy of Sciences, 3888 Eastern South Lake Road, Changchun, 130033, China.

^b^University of Chinese Academy of Sciences, Beijing, 100049, China.

*Corresponding authors. Email: [haozd@ciomp.ac.cn](mailto:haozd@ciomp.ac.cn)**;** zhangjh@ciomp.ac.cn


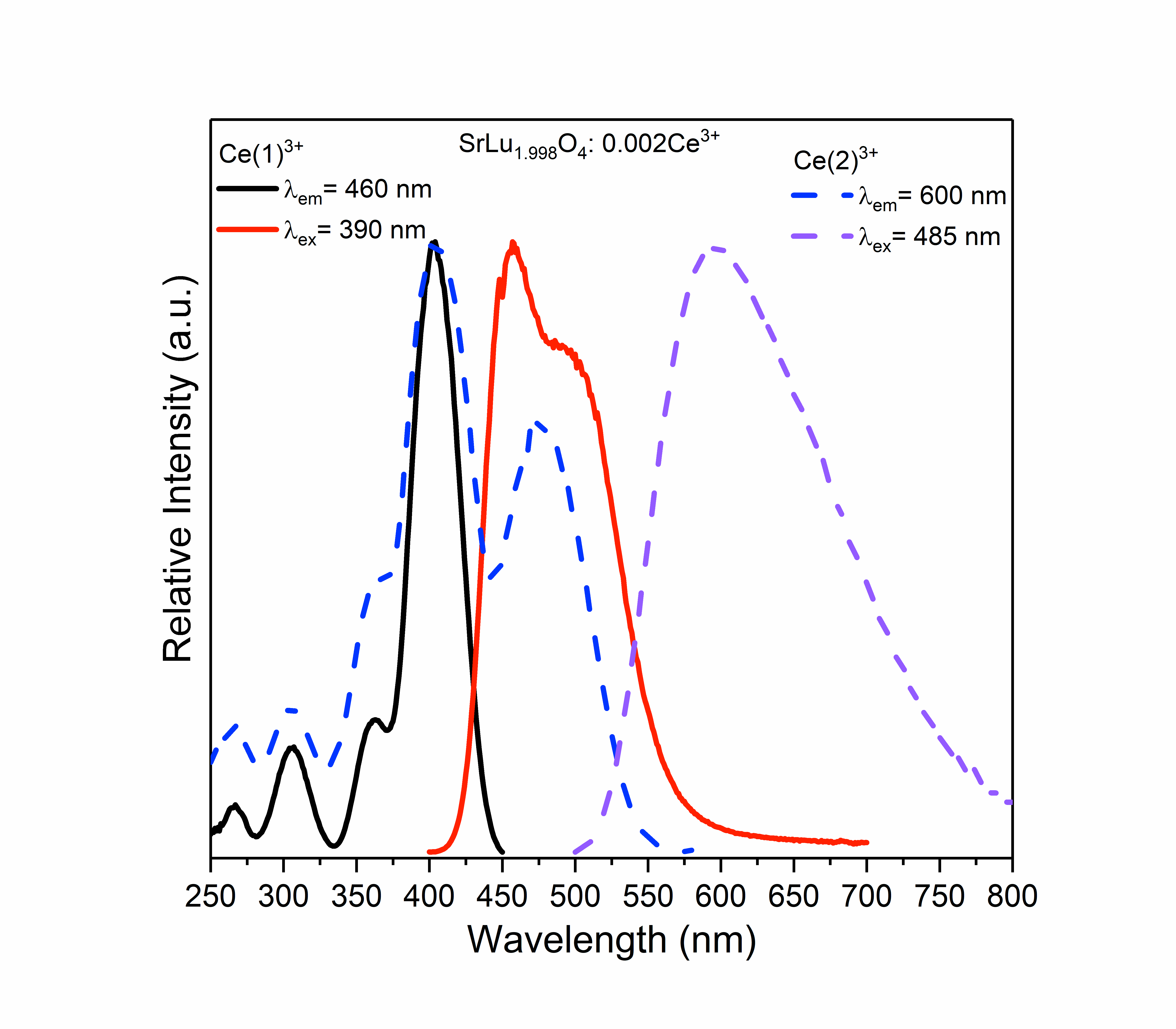


Figure S1: PLE and PL spectra of SrLu_1.998_O_4_: 0.002Ce^3+^ (full lines corresponding to Ce1 and dotted lines corresponding to Ce2).It is obvious that Ce2 center can be excited effectively by the range of both excitation and emission of Ce1 center.


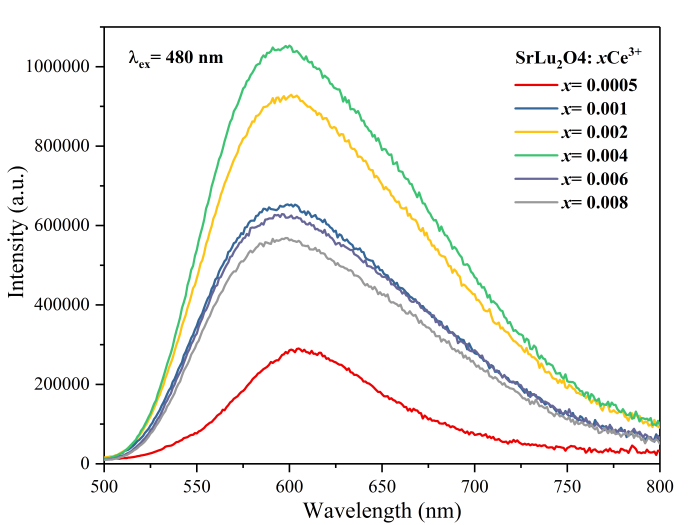


Figure S2: PL spectra of SrLu_2_O_4_: xCe^3+^ (x=0.0005~0.008) under 480-nm excitation.
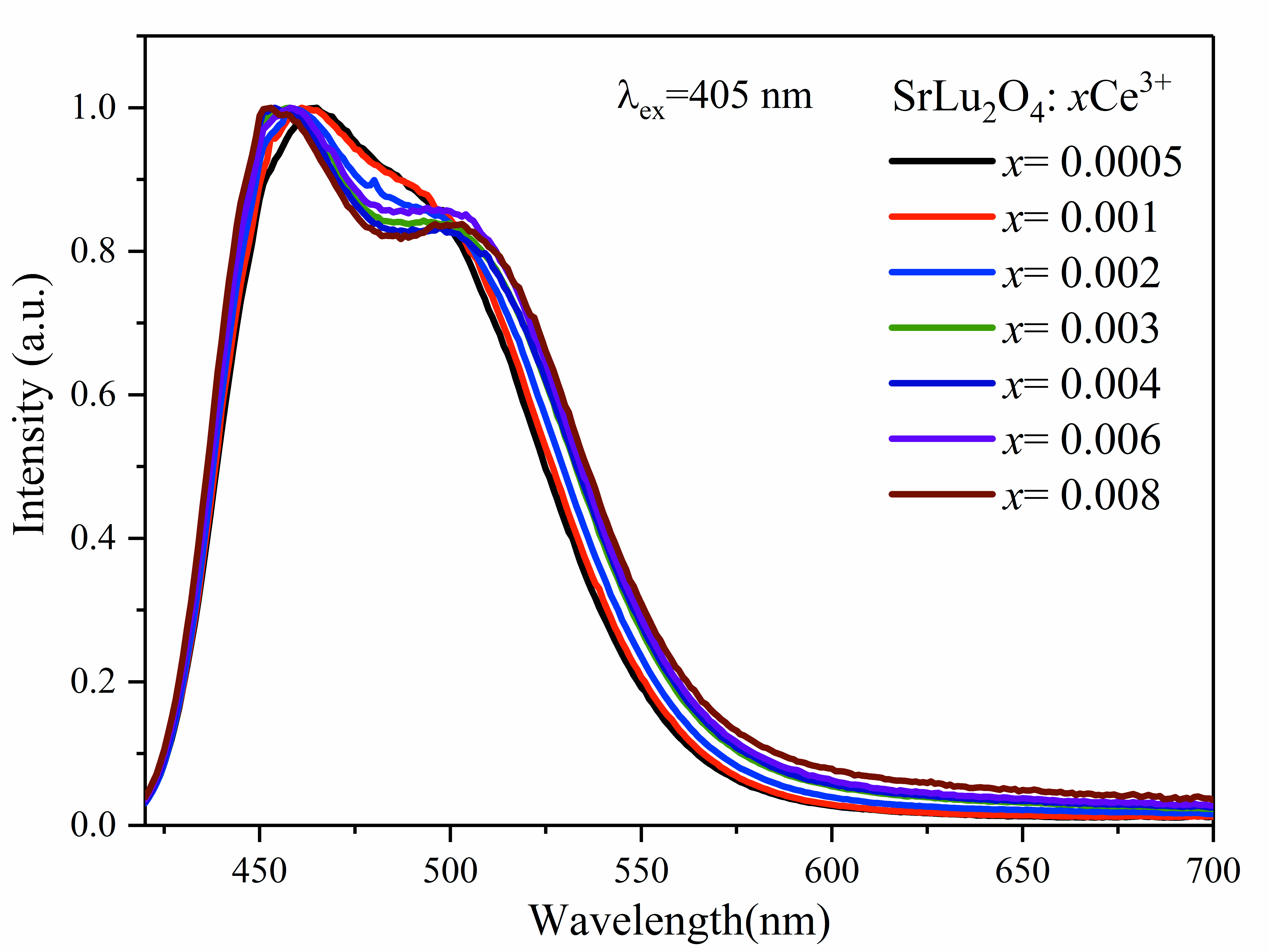


Figure S3: Normalized PL spectra of SrLu_2_O_4_: xCe^3+^ (x=0.0005~0.008) under 405-nm excitation. 600-nm emission enhanced with increasing doping concentration and the intensity is still hard to discernible.


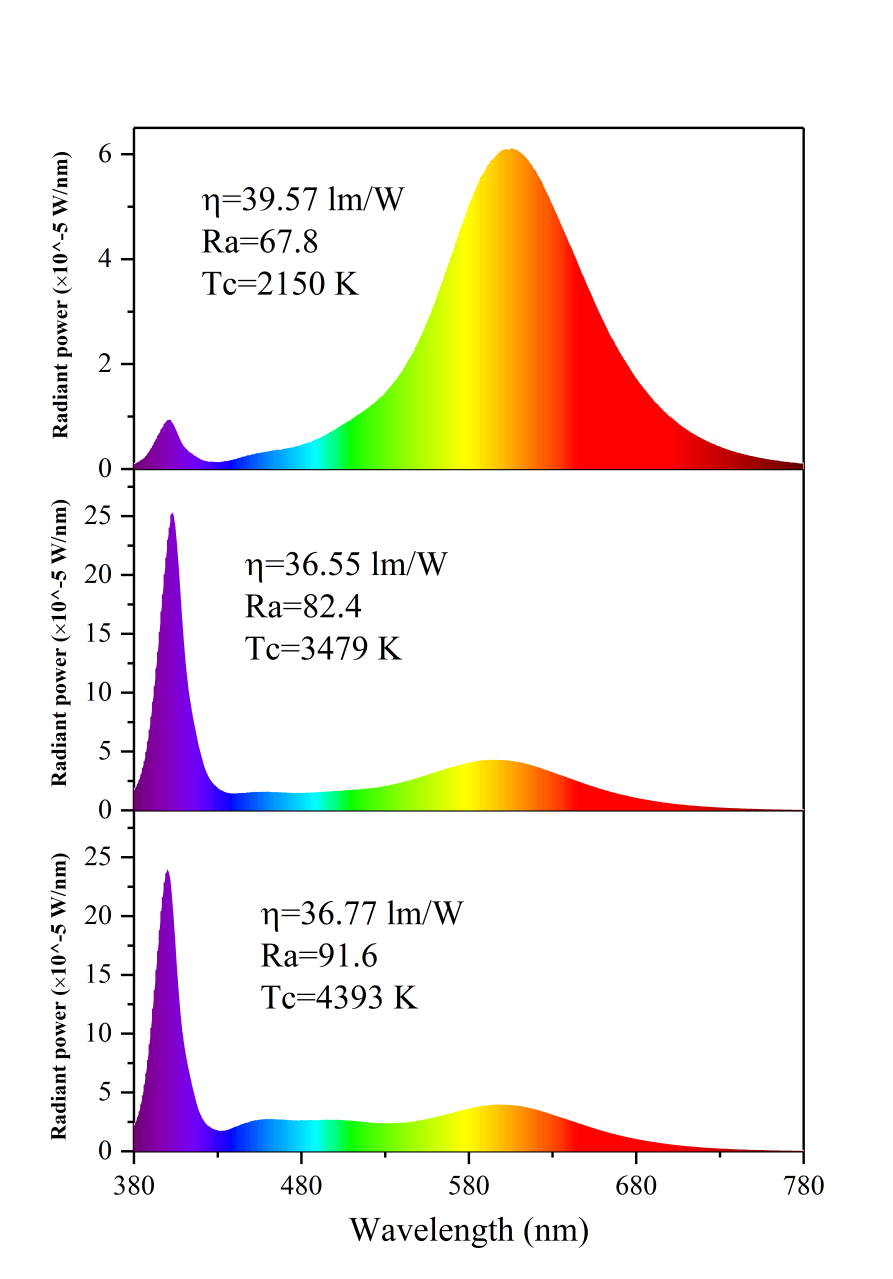


Figure S4: Emission spectra of three white LEDs fabricated using NUV 405-nm chips combined with different ratios of SrLu_2_O_4_: Ce^3+^(blue), (Sr, Ba)_2_SiO_4_: Eu^2+^ (yellow), Sr_2_Si_5_N_8_: Eu^2+^ (red) phosphors and silicone under a forward bias of 20 mA and 3.0 V.


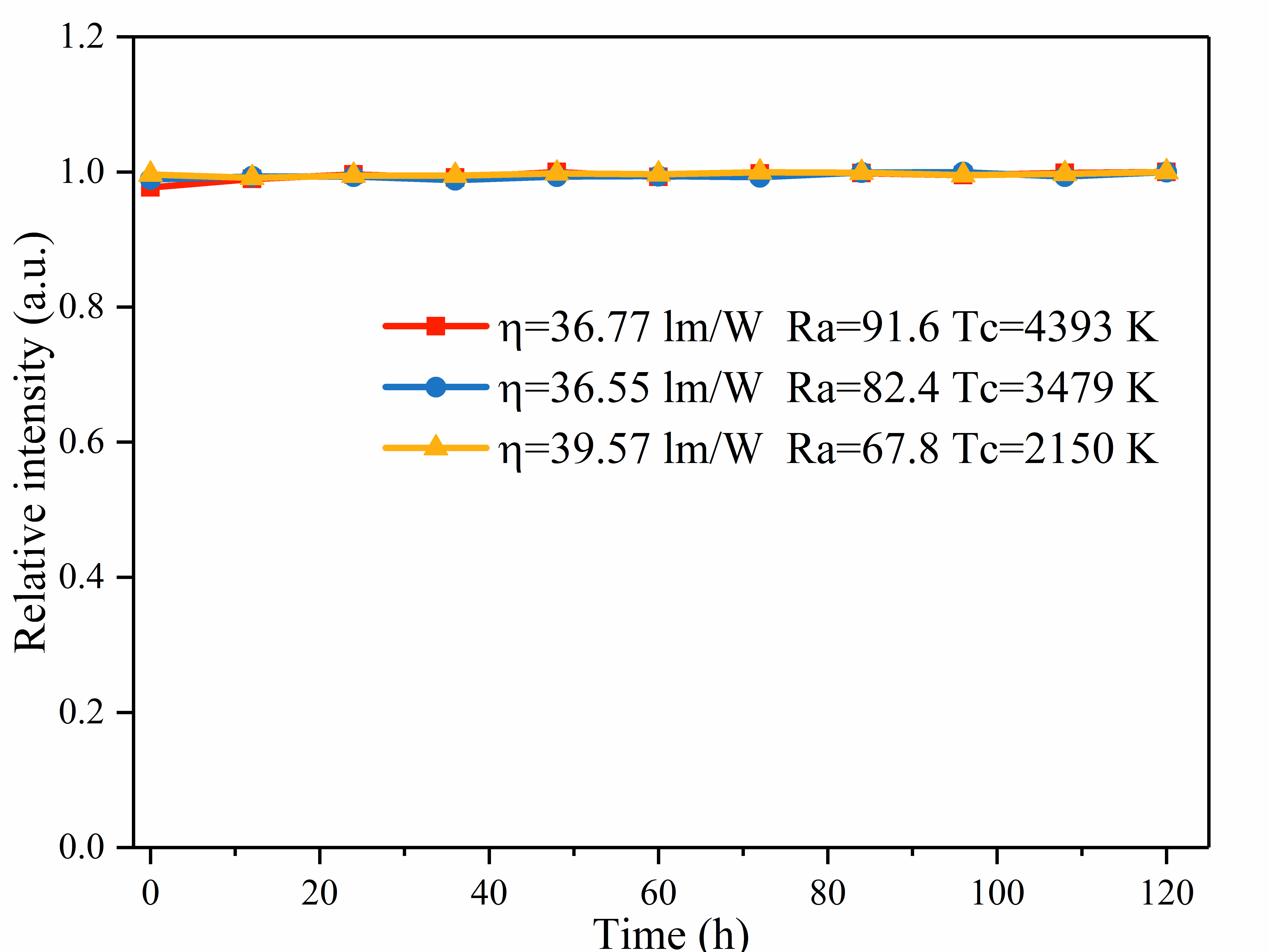


Figure S5: Relative integrated intensities of fabricated white LEDs as a function of time: at temperature of 85℃, in humidity at 85% RH.
